# Whole ovary immunohistochemistry for monitoring cell proliferation and ovulatory wound repair in the mouse

**DOI:** 10.1186/1477-7827-8-98

**Published:** 2010-08-16

**Authors:** Rajasekhar Singavarapu, Natalie Buchinsky, Dong-Joo Cheon, Sandra Orsulic

**Affiliations:** 1 Department of Pathology and Center for Cancer Research, Massachusetts General Hospital, Boston, MA, USA; 2 Women's Cancer Research Institute at the Samuel Oschin Comprehensive Cancer Institute, Cedars-Sinai Medical Center, Los Angeles, CA, USA

## Abstract

**Background:**

Ovarian surface epithelial cells are thought to be a precursor cell type for ovarian carcinoma. It has been proposed that an increased rate of ovarian surface epithelial cell proliferation during ovulatory wound repair contributes to the accumulation of genetic changes and cell transformation. The proliferation of ovarian surface epithelial cells during ovulatory wound repair has been studied primarily using immunohistochemical staining of paraffin-embedded ovary sections. However, such analyses require complex reconstruction from serially-cut ovary sections for the visualization and quantification of the cells on the ovarian surface. In order to directly visualize the proliferation and organization of the ovarian surface epithelial cells, we developed a technique for immunohistochemical staining of whole mouse ovaries. Using this method, we analyzed cell proliferation and morphologic changes in mouse ovarian surface epithelial cells during follicle growth and ovulatory wound repair.

**Methods:**

Three-week old FVB/N female mice were superovulated by sequential administration of pregnant mare's serum gonadotropin (PMSG) and human chorionic gonadotropin (hCG). Ten hours after hCG administration, mice were given 5-bromo-2-deoxyuridine (BrdU) and euthanized two hours after BrdU administration for ovary isolation. The levels of incorporated BrdU in the ovarian surface epithelial cells were measured by staining paraffin-embedded ovary sections and whole ovaries with the BrdU antibody. Re-epithelialization of the ovarian surface after ovulatory rupture was visualized by immunohistochemical staining with E-cadherin and Keratin 8 in paraffin-embedded ovary sections and whole ovaries.

**Results:**

We determined that active proliferation of ovarian epithelial surface cells primarily occurs during antral follicle formation and, to a lesser extent, in response to an ovulatory wound. We also demonstrated that ovarian surface epithelial cells exhibit a circular organization around the wound site

**Conclusion:**

Whole ovary immunohistochemistry enables efficient and comprehensive three-dimensional visualization of ovarian surface epithelial cells without the need for laborious reconstruction from immunohistochemically-stained serial ovary sections.

## Background

Epidemiologic studies show a direct correlation between the number of ovulatory cycles and the risk of ovarian cancer [1-3], suggesting that ovulation may play a role in ovarian carcinogenesis [4]. It is thought that hormone-induced growth of follicles and/or repair of the ovulatory wound result in rapid proliferation of the ovarian surface epithelial cells, which may increase the frequency and accumulation of spontaneous mutations [5,6]. Analyses of cell proliferation and morphologic changes in mouse and rat ovarian surface epithelial cells during different stages of ovulation have been done previously by immunohistochemical staining of paraffin-embedded ovary sections with antibodies against BrdU or proliferating cell nuclear antigen (PCNA) [7-9]. Such analyses are useful for the visualization of ovulation-induced events inside the ovary, however, visualization and quantification of the cells on the ovarian surface require complex three-dimensional reconstruction from serially-cut ovary sections. In order to directly visualize and quantify the proliferating cells on the ovarian surface during different stages of ovulation, we adapted the protocol for immunohistochemistry on slides to whole mouse ovaries.

## Methods

### Superovulation in mice

The use of mice was in accordance with the NIH *Guide for the Care and Use of Laboratory Animals *as well as a protocol approved by the Massachusetts General Hospital Subcommittee on Research Animals (SRAC). The mice were housed with 12 hour light/dark cycle and free access to food and water. Follicle growth and subsequent ovulation in 20 three-week old FVB/N female mice (Charles River Laboratory, Wilmington, MA) were induced by intraperitoneal (i.p.) administration of 5 IU of pregnant mare's serum gonadotropin (PMSG) (Calbiochem, Gibbstown, NJ), followed by i.p. administration of 5 IU of human chorionic gonadotropin (hCG) (Sigma, St. Louis, MO) 46 hours later [10]. The mice were euthanized 12 hours after hCG injection for ovary isolation. Two hours before ovary isolation, 100 mg/kg BrdU (Zymed Laboratories, San Francisco, CA) was administered intraperitoneally into the superovulated mice. Hormone induction typically resulted in 5 to 20 ovulatory sites per ovary. Following bursal removal, the ovaries were isolated, fixed in 10% buffered formalin for 6-12 hours, and randomized for immunohistochemistry on paraffin-embedded sections or whole ovaries.

### Immunohistochemistry

Immunohistochemistry on whole mouse ovaries was done in a 24-well dish that was shaken on a Nutator at room temperature, unless otherwise specified. Fine forceps were used to transfer the formalin-fixed ovaries into a 24-well dish where the ovaries were first washed with phosphate-buffered saline (PBS) then incubated with 3% hydrogen peroxide. For BrdU detection, the ovaries were incubated with hydrogen chloride (2N HCl) for 1 hour, chilled on ice in PBS for 20 minutes, incubated with 0.1% trypsin at 37°C for 20 minutes, and then re-chilled on ice in PBS for 20 minutes (these steps were omitted for E-cadherin and Keratin 8 detection). The ovaries were then transferred into a glass jar with citrate buffer (pH 6.0) (Vector Laboratories, Burlingame, CA) and microwaved for 3 minutes on high power (boiling) and 8 minutes on low power (simmering). After slow cooling for at least 30 minutes, the ovaries were transferred into a 24-well dish on a Nutator and processed for immunohistochemistry with the following peroxidase immunohistochemistry kits from Vector Laboratories: Vector Mouse on Mouse (M.O.M.) for BrdU, Vectastain ABC Rabbit IgG for E-cadherin, and Vectastain ABC Rat IgG for Keratin 8. The ovaries were incubated with the following primary antibodies at room temperature for 30 minutes: mouse monoclonal BrdU (1:100 dilution, Vector Laboratories); rabbit polyclonal E-cadherin (1:100 dilution, Cell Signaling, Danvers, MA); and rat monoclonal TROMA-1 (Keratin 8) (1:25 dilution, Developmental Studies Hybridoma Bank, Iowa City, IA). Bound antibodies were detected by incubating the ovaries with horseradish peroxidase-labeled secondary antibodies (Vector Laboratories) at room temperature for 60 minutes. After color development, the ovaries were washed with PBS. The paraffin-embedded ovary sections were processed for immunohistochemistry in the same manner as the whole ovaries except that the initial steps included deparaffinization and rehydration and the final steps included counterstaining with hematoxylin, dehydration, and mounting with Permount (Fisher Scientific, Pittsburgh, PA). The reproducibility of whole ovary immunohistochemistry was comparable to immunohistochemistry on paraffin-embedded sections; both techniques showed minor day-to-day variations in the intensity of staining. Ovary sections and whole ovaries were visualized using an Olympus BX51 light microscope and an Olympus SZX16 stereomicroscope, respectively (Olympus, Center Valley, PA). The images were captured using a DC-330 3CCD color camera (DAGE-MTI, Michigan City, IN) and adjusted for brightness and contrast using Adobe Photoshop.

### Data analysis

Data were analyzed and presented as dot plots using GraphPad Prism software (GraphPad Software, La Jolla, CA). The statistical significance of differences in the numbers of BrdU positive cells between antral follicles and ovulatory wounds was determined with the Student's *t *test. A value of P < 0.05 was considered to be statistically significant.

## Results

### Detection of epithelial cell proliferation in the ovaries of superovulated mice

Oocyte release during ovulation results in a wound that needs to be repaired by the proliferation of ovarian surface epithelial cells. To better understand and visualize the proliferation of ovarian surface epithelial cells during follicle growth and ovulatory wound repair, we modified the conventional immunohistochemistry protocol for paraffin sections by adapting it to whole ovaries and compared the immunohistochemistry of ovary sections to the whole ovary method by BrdU analysis. For this purpose, 20 three week-old female mice were superovulated by sequential intraperitoneal administration of PMSG and hCG [10], and pulsed for 2 hours with BrdU. The ovaries were isolated 12 hours after hCG administration because this is the time when ovulation is expected to occur [10]. Hormone induction typically resulted in 5 to 20 ovulatory sites per ovary. Twenty ovaries were processed as paraffin sections and 20 were used for whole ovary immunohistochemistry.

**Figure 1:**
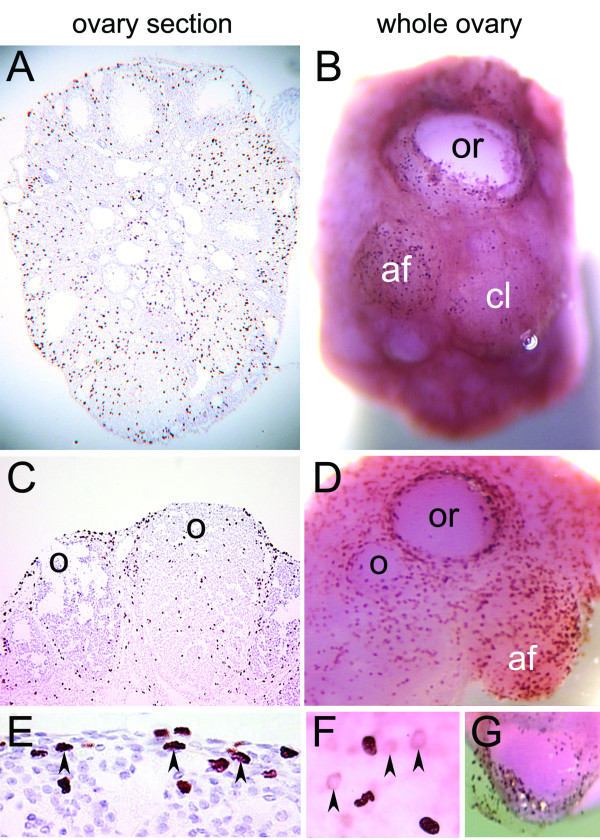
**BrdU staining of ovary sections and whole ovaries in superovulated mice**. BrdU was administered to the mice 10 hours after hCG injection and the ovaries were isolated 2 hours after BrdU injection. The distribution of cells with incorporated BrdU was determined by staining paraffin-embedded ovary sections (A, C, E) and whole ovaries (B, D, F, G) with anti-BrdU antibody. Arrowheads indicate BrdU positive cells below the ovarian surface epithelial layer. Abbreviations: af, antral follicle; cl, corpus luteum; o, ovulatory wound; or, recent ovulatory wound.

We monitored proliferation of the ovarian surface epithelial cells using nuclear incorporation of BrdU over a two-hour period in the ovary sections (Figure 1A and 1C) and the whole ovaries (Figure 1B and 1D). In ovary sections, the BrdU signal was detected in the surface epithelial cells as well as in the underlying stromal, follicular, and luteal cells (Figure 1A and 1C), including the cells that are one layer beneath the ovarian surface (arrowheads in Figure 1E). In the whole ovaries, the staining typically consisted of well-defined and strong BrdU signals (Figure 1B and 1D), although weak and blurry staining was also detectable in some ovaries under high magnification (arrowheads in Figure 1F). The distribution of BrdU positive cells in the surface epithelia was more easily assessed in the whole ovaries than in the ovary sections. The greatest number of BrdU positive cells was observed in growing antral follicles (af), edges of recent ovulatory wounds (or), and corpora lutea containing ovulatory wounds in the process of repair (o) (Figure 1B and 1D). Less BrdU positive cells were detected in fully repaired corpora lutea (cl) (Figure 1B). To determine the focal depth of the BrdU labeling in the whole ovaries, we pealed off a patch of epithelial layer after the staining. The BrdU antibody penetration was largely confined to the epithelial layer while a weak signal was present in the underlying cells (arrowheads in Figure 1G).

**Figure 2:**
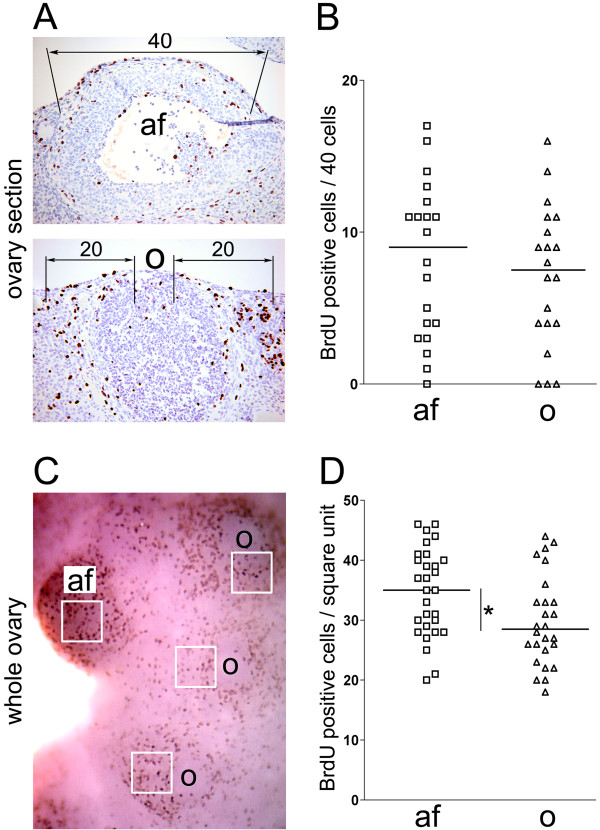
**Quantification of BrdU incorporation during antral follicle formation and ovulatory wound repair in ovary sections (A, B) and whole ovaries (C, D)**. (A) In ovary sections, the number of BrdU positive cells associated with the antral follicles (af) was determined by scoring forty cells covering the surface of each antral follicle. The number of BrdU positive cells associated with corpora lutea containing healing ovulatory wounds (o) was determined by scoring twenty cells covering each side of the ovulatory wound. (B) Dot plot of the number of BrdU positive cells associated with antral follicles and ovulatory wounds. Each rectangle represents one antral follicle (N = 20) and each triangle represents one corpus luteum with an ovulatory wound (N = 20). The horizontal bars represent the mean values. P = 0.5026. (C) In the whole ovary, the number of BrdU positive cells associated with antral follicles (af) and ovulatory wounds (o) was determined by counting positive cells per square unit. (D) Dot plot of the number of BrdU positive cells associated with antral follicles (af) and ovulatory wounds (o). Each rectangle represents one antral follicle (N = 29) and each triangle represents one corpus luteum with an ovulatory wound (N = 26). The horizontal bars represent the mean values. *P = 0.0175.

In order to compare the proliferation events of ovarian surface epithelial cells observed from two different methods, we quantified BrdU-positive cells covering the antral follicles (af) and the corpora lutea with ovulatory wounds (o) in the ovary sections (Figure 2A and 2B) and the whole ovaries (Figure 2C and 2D). In the ovary sections, 20 antral follicles (af) and 20 ovulatory wounds (o) were scored for BrdU positive cells. The number of BrdU positive cells was determined by counting 40 cells on top of each antral follicle and 20 cells surrounding each ovulatory rupture site on both sides (Figure 2A). The number of BrdU positive cells covering the antral follicles ranged from 0 to 17 (mean = 8.15; standard deviation = 5.14), while the number of BrdU positive cells surrounding the ovulatory wounds ranged from 0 to 16 (mean = 7.1; standard deviation = 4.66) (Figure 2B). A slightly higher proliferation rate was observed in the antral follicles than in the ovulatory wounds, although this difference was not statistically significant (P = 0.5026). In whole ovaries immunostained with BrdU, the number of BrdU positive cells per square unit in the antral follicles (af) and the ovulatory wounds (o) was determined as shown in Figure 2C. Twenty nine antral follicles and 26 ovulatory wounds were scored. The numbers of BrdU positive cells per square unit ranged from 21 to 46 (mean = 34.72; standard deviation = 7.34) in the antral follicles and from 18 to 44 (mean = 29.8; standard deviation = 7.52) in the ovulatory wounds (Figure 2D). Interestingly, whole ovary immunohistochemitry showed a statistically significant (P = 0.0175) higher number of proliferating cells in the antral follicles than in the ovulatory wounds.

### Re-epithelialization of the ovulatory wound

**Figure 3:**
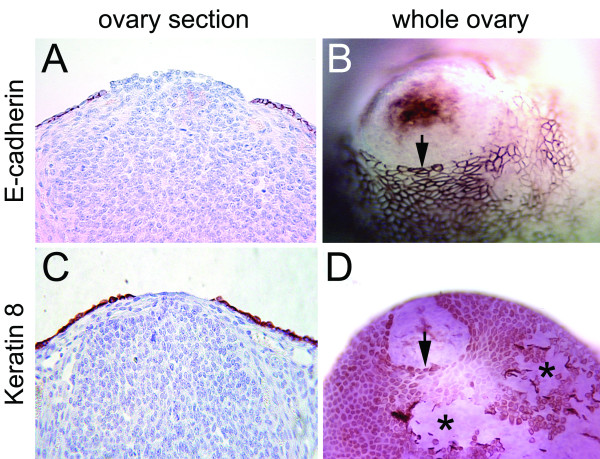
**Re-epithelialization of the ovarian surface after ovulatory rupture**. Ovarian surface epithelial cells were visualized by immunohistochemical staining with E-cadherin (A, B) and Keratin 8 (C, D) in paraffin-embedded ovary sections (A, C) and whole ovaries (B, D). The arrows point to well-organized epithelial edges around the ovulatory wound. The asterisks indicate disorganized epithelia where the ovarian surface was artificially scraped by ovary manipulation.

In order to follow re-epithelialization of the ovarian surface after follicular rupture, we labeled cells with the epithelial cell markers, E-cadherin and Keratin 8, using ovary sections (Figure 3A and 3C) or whole ovaries (Figure 3B and 3D). In the ovary sections, strong signals of E-cadherin and Keratin-8 were detected in ovarian surface epithelia (Figure 3A, C). In the whole ovaries, surface epithelial cells also showed positive staining of both E-cadherin and Keratin-8. E-cadherin showed variable levels of epithelial cell membrane expression (Figure 3B), while Keratin-8 was evenly expressed in the cytoplasm of the ovarian surface epithelial cells (Figure 3D). Whole ovary immunohistochemistry revealed a circular organization of ovarian surface epithelial cells around the ovulatory wound (arrows in Figure 3B and 3D) in contrast to the disorganized epithelia where the ovarian surface was artificially scraped by ovary manipulation (asterisks in Figure 3D).

**Figure 4:**
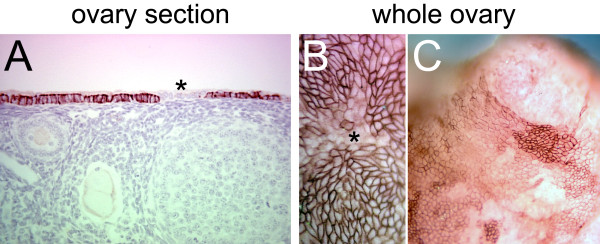
**E-cadherin expression in the ovarian surface epithelial cells**. Immunohistochemical staining for E-cadherin in a paraffin-embedded ovary section (A) and whole ovaries (B, C). The asterisks indicate groups of epithelial cells in which E-cadherin is expressed at a low level. Distinct patches of ovarian surface epithelial cells with variable levels of E-cadherin expression are detected by whole ovary immunohistochemistry (C).

Low or nonexistent expression of E-cadherin was observed in some cells in the contiguous ovarian surface epithelial layer in the paraffin sections (Figure 4A) but it was unclear if these cells were organized in discrete patches. Immunohistochemistry on whole ovaries revealed that the ovarian surface epithelial cells with variable levels of E-cadherin expression were organized into distinct patches (Figure 4B and 4C). The functional significance of these patches of ovarian surface epithelial cells with different expression of E-cadherin is presently unknown.

## Discussion

Most studies on the proliferation of ovarian surface epithelial cells during ovulatory follicle growth and postovulatory wound repair rely on the immunohistochemistry of paraffin sections of the ovaries, which lack information on the spatial distribution of proliferating ovarian surface epithelial cells. To better visualize the proliferation of the ovarian surface epithelial cells during follicle growth and ovulatory wound repair, we applied a modified immunohistochemistry protocol to whole ovaries and compared our method to conventional immunohistochemistry on paraffin sections. Using ovary sections and whole ovaries, we determined that ovarian surface cell proliferation is more active during antral follicle growth than during ovulatory wound repair. Our results are consistent with previous reports in which paraffin-embedded sections of hormone-stimulated mouse ovaries and unstimulated rat ovaries were used to determine the rate of cell proliferation in discrete anatomical regions of the ovary [7-9]. One disadvantage of whole ovary immunohistochemistry in comparison to conventional immunohistochemistry on the ovary sections was in the inability to visualize BrdU negative cells on the ovarian surface. However, it is likely that the use of hematoxylin as a contrasting agent in whole ovaries would allow for the visualization of BrdU negative cells in a similar manner as in conventional immunohistochemistry on ovary sections.

Whole ovary immunohistochemistry was particularly useful for the visualization of epithelial cell organization during ovulatory wound repair. We determined that epithelial cells are organized in a circular pattern around the healing ovulatory wound. Moreover, we detected distinct patches of cells that express different levels of E-cadherin. It is presently unclear why the cells that express different levels of E-cadherin are organized into patches and whether these patches play a specific role in ovulatory wound repair.

Although whole mount immunohistochemistry is commonly used with embryo or organ specimens in *Drosophila *and *Xenopus*, its use in murine research has been limited to the study of embryogenesis [11-13] and brain development [14-16]. The advantage of using whole ovary immunohistochemistry for the quantification of ovarian surface epithelial cell proliferation and the visualization of ovarian surface re-epithelialization after follicular rupture is the ability to directly visualize the entire surface of the ovary, without the need to reconstruct the surface from serial sections. This technique could be easily adapted for whole ovary immunohistochemistry for other species, including human.

## Competing interests

The authors declare that they have no competing interests.

## Authors' contributions

RS and NB designed and performed the experiments. D-JC interpreted the data and participated in the writing of the manuscript. SO conceived and supervised the study and wrote the manuscript. All authors read and approved the final manuscript.
